# HPVdb: a data mining system for knowledge discovery in human papillomavirus with applications in T cell immunology and vaccinology

**DOI:** 10.1093/database/bau031

**Published:** 2014-04-04

**Authors:** Guang Lan Zhang, Angelika B. Riemer, Derin B. Keskin, Lou Chitkushev, Ellis L. Reinherz, Vladimir Brusic

**Affiliations:** ^1^Cancer Vaccine Center, Dana-Farber Cancer Institute, 77 Ave Louis Pasteur, Boston, MA 02115, USA, ^2^Department of Computer Science, Metropolitan College, Boston University, 808 Commonwealth Ave, Boston, MA 02215, USA, ^3^Department of Medicine, Harvard Medical School, 25 Shattuck Street, Boston, MA 02115, USA and ^4^German Cancer Research Center (DKFZ), Im Neuenheimer Feld 280, 69120 Heidelberg, Germany

## Abstract

High-risk human papillomaviruses (HPVs) are the causes of many cancers, including cervical, anal, vulvar, vaginal, penile and oropharyngeal. To facilitate diagnosis, prognosis and characterization of these cancers, it is necessary to make full use of the immunological data on HPV available through publications, technical reports and databases. These data vary in granularity, quality and complexity. The extraction of knowledge from the vast amount of immunological data using data mining techniques remains a challenging task. To support integration of data and knowledge in virology and vaccinology, we developed a framework called KB-builder to streamline the development and deployment of web-accessible immunological knowledge systems. The framework consists of seven major functional modules, each facilitating a specific aspect of the knowledgebase construction process. Using KB-builder, we constructed the Human Papillomavirus T cell Antigen Database (HPVdb). It contains 2781 curated antigen entries of antigenic proteins derived from 18 genotypes of high-risk HPV and 18 genotypes of low-risk HPV. The HPVdb also catalogs 191 verified T cell epitopes and 45 verified human leukocyte antigen (HLA) ligands. Primary amino acid sequences of HPV antigens were collected and annotated from the UniProtKB. T cell epitopes and HLA ligands were collected from data mining of scientific literature and databases. The data were subject to extensive quality control (redundancy elimination, error detection and vocabulary consolidation). A set of computational tools for an in-depth analysis, such as sequence comparison using BLAST search, multiple alignments of antigens, classification of HPV types based on cancer risk, T cell epitope/HLA ligand visualization, T cell epitope/HLA ligand conservation analysis and sequence variability analysis, has been integrated within the HPVdb. Predicted Class I and Class II HLA binding peptides for 15 common HLA alleles are included in this database as putative targets. HPVdb is a knowledge-based system that integrates curated data and information with tailored analysis tools to facilitate data mining for HPV vaccinology and immunology. To our best knowledge, HPVdb is a unique data source providing a comprehensive list of HPV antigens and peptides.

**Database URL:** http://cvc.dfci.harvard.edu/hpv/

## Introduction

Papillomaviruses are small double-stranded DNA viruses that infect the squamous epithelia (skin and internal mucosae) of both animals and humans (1). Papillomaviruses are diverse and species-specific. The human papillomavirus (HPV) genome is composed of six early (E1, E2, E4, E5, E6 and E7) Open Reading Frames (ORFs), two late (L1 and L2) ORFs and a non-coding long control region (2). More than 170 HPV types have been characterized to date. Of those, nearly 40 are transmitted through sexual contact, infecting the anogenital region and the oropharynx. Among these, ∼20 are designated as ‘oncogenic high risk’ because they have been linked to cervical, anal, vulvar, vaginal, penile and oropharyngeal cancers (3). Worldwide, >5% of all new cancers are attributed to high-risk HPV infections (4). HPV, Hepatitis B virus, Hepatitis C virus and *Helicobacter pylori* were responsible for 1.9 million cancer cases worldwide in 2008 (5). HPV is the cause of virtually all cases of cervical cancer, the third most common female cancer globally (6). The high-risk type HPV16 alone is responsible for 50% of cervical cancers and high-grade cervical intraepithelial lesions (7). Furthermore, in the developing world where HPV disease burden is the greatest, cervical carcinoma is the leading cause of cancer mortality among women.

Two prophylactic HPV L1 VLP (virus-like particle) vaccines have been developed to provide protection against infection for at least 5 years and reduce the risk of cervical cancer (8). Gardasil, an HPV quadrivalent recombinant vaccine that is a mixture of VLPs derived from the L1 capsid proteins of HPV types 6, 11, 16 and 18, was approved by the US Food and Drug Administration in 2006 (9). Cervarix, a prophylactic vaccine composed of a mixture of VLPs derived from the L1 capsid proteins of HPV types 16 and 18 has been shown to be 100% effective in preventing HPV strains 16 and 18 (10). T cells naturally eliminate the majority of HPV infections by recognizing epitopes displayed on the virally altered epithelium. Therapeutic HPV vaccines aim to treat established HPV infections and HPV-associated malignancies by targeting non-structural oncogenic proteins E6 and E7 (11). A recent vulvar intraepithelial neoplasia clinical trial showed promising results. Kenter *et al.* (12) reported a vaccination using synthetic long peptides spanning the complete sequence of the HPV16 E6 and E7 oncoproteins and a conventional adjuvant-induced clinical responses and relief of symptoms in 60% of the patients with high-grade vulval intraepithelial neoplastic disease. However, most trials of therapeutic HPV vaccines have yielded disappointing clinical results (11).

Available immunological data related to HPV vary in granularity, quality and complexity and are stored in various formats, from publications, technical reports and general-purpose databases. The challenge is to collect the data scattered in these resources, clean, annotate, store and analyse them to facilitate meaningful knowledge discovery. To bridge the gap between data and knowledge, we developed a framework called KB-builder to streamline the development and deployment of web-accessible immunological knowledge bases. The KB-builder framework is generic and can be applied to any immunological sequence data set. We developed the Human Papillomavirus T cell Antigen Database (HPVdb) using KB-builder to support the discovery of T cell-based HPV vaccine targets and reported it at the Association for Computing Machinery - Conference on Bioinformatics, Computational Biology and Biomedical Informatics (ACM-BCB) 2013 (13). We updated and improved HPVdb by expanding the data sets and integrating more advanced analysis tools such as antigen sequence variability analysis tool based on Shannon entropy calculation and conservation analysis of T cell epitopes and human leukocyte antigen (HLA) ligands. HPVdb integrates curated data and information with tailored analysis tools to facilitate data mining and to aid rational vaccine design by discovery of vaccine targets. It is publicly available at http://cvc.dfci.harvard.edu/hpv/.

## Material and Method

### KB-builder

**Figure 1.**
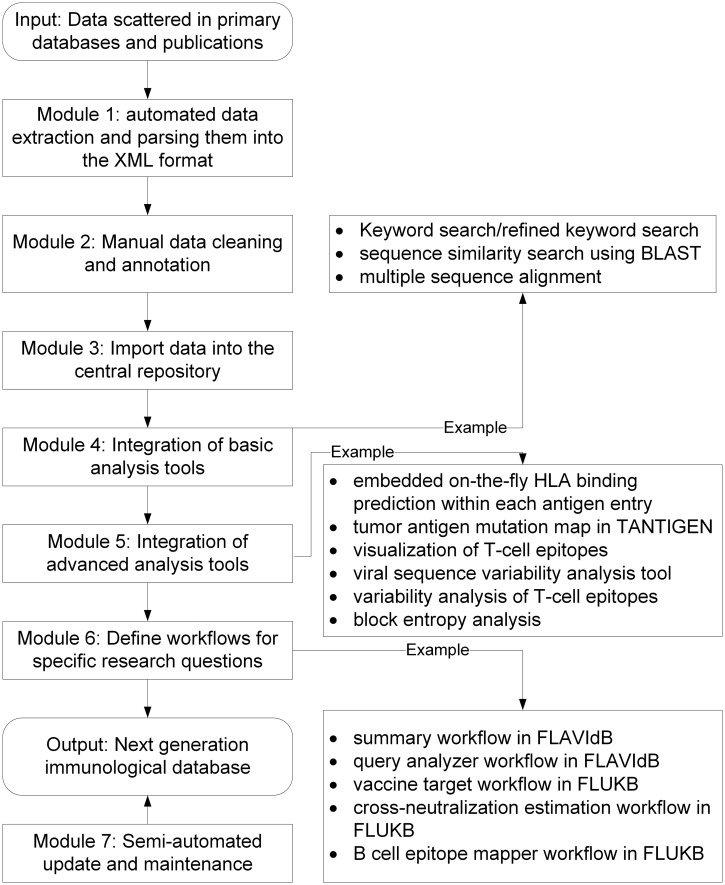
Schematic overview of the KB-builder framework.

The framework, KB-builder, consists of seven major functional modules, each facilitating a specific aspect of the database construction process. The input to the framework is data scattered in primary databases and scientific literatures. As shown in Figure 1, the modules enable automated data collection and integration, semi-automated data cleaning and annotation, automated data storage and retrieval, fast deployment of basic computational tools, development and integration of advanced tools for an in-depth analysis of various structural and functional properties associated with immune responses and vaccine development, definition of workflows to answer specific research questions and semi-automated update and maintenance. The KB-builder framework helps set up a web-accessible knowledgebase and the corresponding analysis pipeline within a short period (typically within 1–2 weeks), given a set of annotated genetic or protein sequences. In addition to the HPVdb, using KB-builder, several other prototype knowledge bases have been built including TANTIGEN: Tumor T cell Antigen Database (cvc.dfci.harvard.edu/tadb), FLAVIdB: Flavivirus Antigen Database (14) and FLUKB: Flu Virus Antigen Database (research4.dfci.harvard.edu/cvc/flukb/). A workflow is an automated process that takes a request from the user, performs complex analysis by combining data and tools preselected for common questions and produces a comprehensive report (15). Several workflows have been implemented in FLAVIdB and FLUKB to answer various research questions, such as the identification of broadly protective viral vaccine targets (14). The web interface of these online knowledge bases uses a set of graphical user interface forms with a combination of Perl, PHP, CGI and C background programs. Development was carried out in the CentOS 4.5 Linux environment.

### Data collection

Eight HPV proteins are included in the HPVdb, namely, E1, E2, E4, E5, E6, E7, L1 and L2. Eighteen HPV genotypes (16, 31, 33, 35, 52, 58, 18, 39, 45, 59, 68, 26, 51, 82, 73, 53, 56 and 66) of five HPV species (HPV16, HPV18, HPV26, HPV34 and HPV53) were annotated as the high-risk group in HPVdb. Eighteen HPV genotypes (11, 44, 55, 40, 91, 32, 42, 54, 61, 72, 81, 83, 84, 62, 87, 89, 71 and 64) of six HPV species (HPV6, HPV7, HPV32, HPV54, HPV61, HPV71 and one unclassified species) were classified as the low-risk group. Names, full names, virus genotypes, UniProt review status, sequence status and primary amino acid sequences of HPV antigens from the aforementioned 36 genotypes were collected from the UniProtKB (16). Primary amino acid sequences of HPV were collected via database search against the UniProtKB using the NCBI taxonomic identifiers of the 18 HPV genotypes, for example, 333 760 is the taxonomic identifier of organism ‘HPV type 16’.

HPV T cell antigens were collected based on experimentally characterized T cell epitopes and/or HLA ligands. The criteria for the selection of HPV T cell antigens require that the antigen must be presented via one or more HLA alleles or be recognized by T cells. If the peptides were able to stimulate T cell function, they are called ‘T cell epitopes’. If the peptides had only been tested for their binding affinities to HLA molecules and not for T cell reactivity, these peptides are called ‘HLA ligands’. Collection of T cell epitopes and HLA ligands was derived from mining of scientific literature in PubMed using the term ‘HPV AND (T cell epitope OR HLA binding peptide)’ as a keyword and from the Immune Epitope Database (IEDB) (17).

### Data annotation and organization

The collected data and information were manually checked. Errors, inconsistencies, ambiguous and conflicting information and duplications were corrected or removed. Sequences containing ambiguous amino acids, such as XGXXNGILW, were removed because they make it impossible to perform computational prediction for HLA binding motifs. If multiple UniProt entries have identical sequences, only one entry is kept. If a UniProt entry sequence is a substring of a longer sequence, the entry is removed and the entry with longer sequence is kept. The semi-structured annotated data were automatically transformed in a unified extensible markup language (XML) format because XML is inherently semi-structured and is suitable for hosting semi-structured data. Three XML files were created for HPVdb containing information on antigens, T cell epitopes and HLA ligands. Information in an HPV antigen record includes antigen name, full name, virus genotype, UniProt ID, UniProt review status, amino acid sequences, as well as T cell epitopes and HLA ligands, if reported.

### Data classification

**Figure 2.**
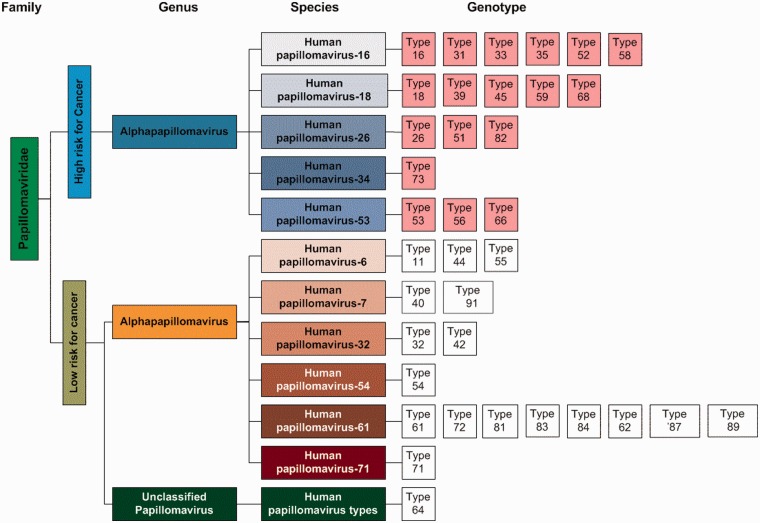
Classification of the viruses in the HPVdb based on cancer risk was done using the virus classification system suggested by the ICTV.

In HPVdb, HPV viruses were classified into two groups based on high and low clinical risk for cancer. Each risk group was further subclassified using the virus classification system suggested by the International Committee on Taxonomy of Virus (ICTV). An interactive diagram presents the classification and facilitates database search by clicking on any of the genotype boxes (Figure 2).

### Database construction

The HPVdb was constructed using KB-builder, an in-house developed framework that streamlines the development and deployment of web-accessible immunological knowledge bases. The web interface uses a set of graphical user interface forms with a combination of Perl, PHP, Common Gateway Interface (CGI) and C background programs.

### Basic analysis tools

Several basic bioinformatics tools were integrated in the HPVdb, including a keyword search tool to locate HPV antigens, a keyword search tool to locate the T cell epitopes or HLA ligands of interest and Basic Local Alignment Search Tool (BLAST) that enables sequence similarity search (18) and multiple sequence alignment (MSA) to compare multiple sequences. Beyond the basic utility of keyword search, the HPVdb also offers options for filtering HPV antigen data based on genotype, proteins, UniProt review status (reviewed or unreviewed) and sequence type (complete or fragment sequence). To search for T cell epitopes or HLA ligands, users may input either an epitope/ligand sequence or an HLA allele of interest in the text box. If nothing is input into the text box, the search result page will show all the T cell epitopes and HLA ligands in the database. To facilitate sequence similarity search, the collected antigen protein sequences were organized into FASTA format and were converted into a searchable format to enable searching using the Basic Local Alignment Search Tool (BLAST) algorithm (18). MAFFT, an MSA tool, selected because of its outstanding performance in terms of speed and alignment quality, was downloaded and installed locally (19).

### Specialized analysis tools

The HLA binding prediction tool for on-the-fly peptide binding prediction to 15 frequent HLA class I and class II alleles (A*0101, A*0201, A*0301, A*1101, A*2402, B*0702, B*0801, B*1501, DRB1*0101, DRB1*0301, DRB1*0401, DRB1*0701, DRB1*1101, DRB1*1301, DRB1*1501) has been integrated in each HPV antigen record to facilitate efficient antigenicity analysis. NetMHCpan and NetMHCIIpan (20, 21) were selected for this purpose based on our previous benchmark studies on the accuracy of online HLA binding prediction servers (22, 23). Other data mining tools include the sequence variability analysis tool, the conservation analysis tool for T cell epitopes and HLA ligands and the visualization tool that shows the localization of epitopes in a given individual or aligned HPV protein/genotype combination. Sequence variability analysis can be performed on entries grouped by protein and further narrowed down by virus genotype or subtype and sequence type. The variability analysis at amino acid level is based on calculation of Shannon entropy (24) at each position in an MSA. The entropy is calculated using the formula
(1)
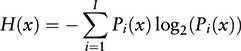

where *H* is the entropy, *x* is the position in the MSA, *i* represents individual amino acids at position *x*, *I* is the number of different amino acids on position *x* and *P_i_* is the frequency of the given amino acid. Conservation of a position, *x*, is defined by the frequency of the consensus amino acid.

### Semiautomated update and maintenance

The database will be actively maintained by members of the bioinformatics core, cancer vaccine center, the Dana-Farber Cancer Institute. Bugs and problems will be fixed as they are reported. The database will be updated every 6 months, using automated retrieval systems such as Wget (http://www.gnu.org/software/wget/) and data from primary databases such as the Uniprot and the IEDB. Programs have been made to automatically compare the collected data with the existing data in the database and identify new data; the new data will be manually annotated; and the XML data files will be automatically updated to include the annotated new data. On completion of the data update, the last step of the database update is to test all the basic and advanced analysis tools and workflows to ensure they function well with the updated data files.

## Results

Using the NCBI taxonomic identifiers of the 18 HPV genotypes to search the UniProtKB, we collected 5099 antigen entries (as of December 2013), of which 162 entries were reviewed by UniProt experts and 4937 entries were not reviewed. The data cleaning process removed 2318 antigen entries—5 entries had unknown gene names such as ‘X’ (UniProt ID: Q705E1) and ‘Y’ (UniProt ID: Q705E0), 83 antigen sequences contained ambiguous amino acid X and 2230 had redundant sequences. The final list has 2781 antigen entries (Table 1). Eight HPV proteins were included in the HPVdb, namely, E1, E2, E4, E5, E6, E7, L1 and L2. A large number of antigen sequences have been reported for proteins E1 (630), E2 (320), E6 (419), E7 (233), L1 (624) and L2 (296). Around one-third of these sequences were derived from species HPV16 (978). We observed that inconsistent letter case has been used in gene names. For example, gene names e7 (UniProt ID: Q9DIH6) and I2 (UniProt ID: Q9DHD4) were listed instead of E7 and L2. All lower case gene names were modified to upper case. In total, 191 verified T cell epitopes and 45 verified HLA ligands were collected from data mining of literature and databases.

**Table 1.** The number of antigen entries in HPVdb grouped by their UniProt review status and type of antigen sequences

Sequence Type	Reviewed	Not reviewed	Total
Complete sequence	160	1684	1844
Fragment	2	935	937
Total	162	2619	2781

**Figure 3.**
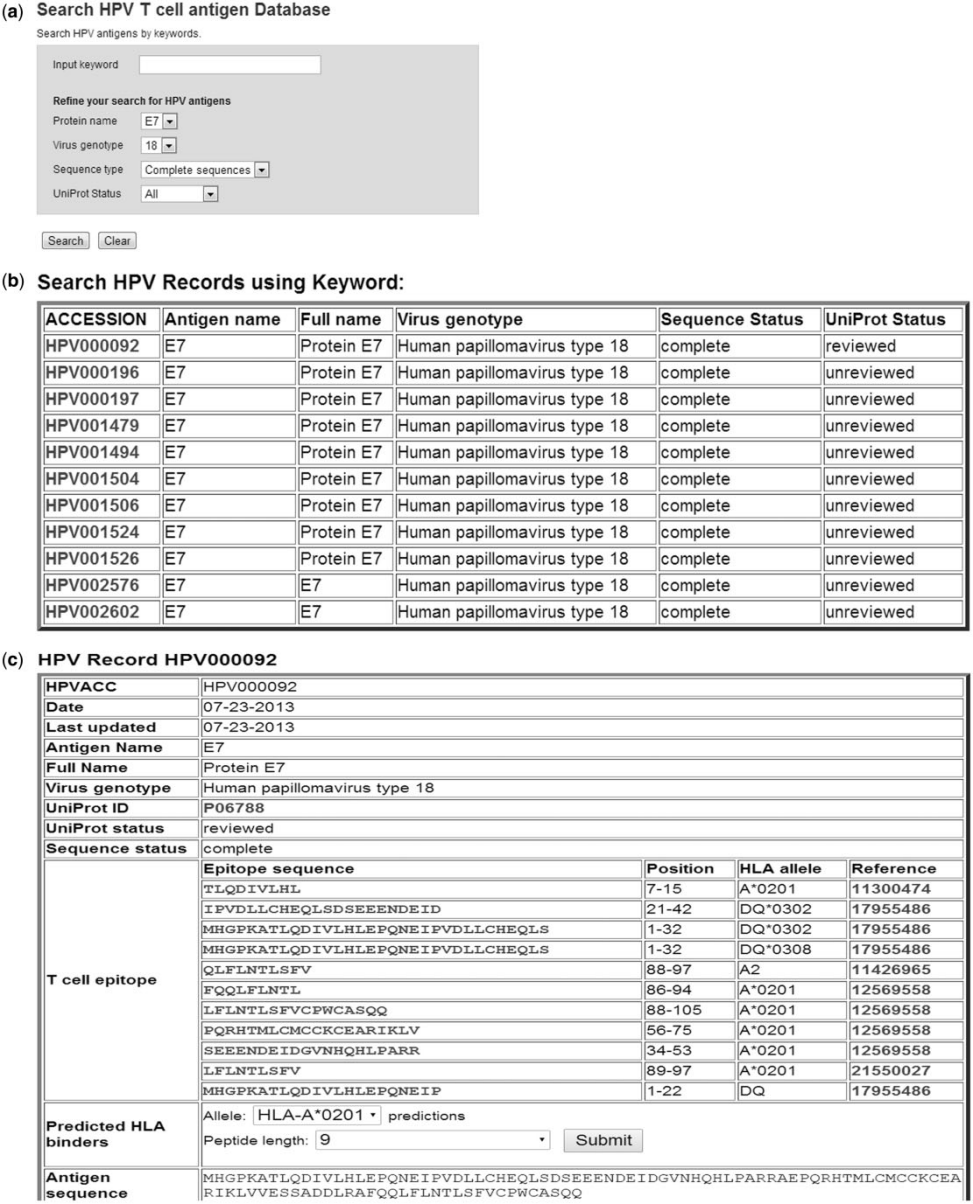
Screenshots of HPV antigen search tool and result pages. (**A**) HPV antigen search page. (**B**) The search result page—the accession numbers in the result table are hyperlinked to HPV antigen information pages. (**C**) HPV00092 (UniPort ID: P06788) information table.

Using the keyword search function of HPVdb, users are able to search antigen records by keywords. Users can further refine the search by selecting a protein name, and/or a virus genotype, and/or a sequence type and/or UniPort review status. Figure 3A shows the HPVdb antigen search page with parameters selected being Protein E7, virus genotype 18, complete sequence regardless of UniProt review status. Figure 3B shows the search result table. The accession numbers in the first column are hyperlinked. By clicking on HPV000092, we get a page, as shown in Figure 3C, displaying information on the antigen. The antigen information table consists of HPVACC (a unique accession number), date, last updated date, antigen name, full name, virus genotype, UniProt ID, UniProt status (reviewed or unreviewed), sequence status (complete or fragment), a list of T cell epitopes and HLA ligands of the antigen with references, antigen amino acid sequence and the embedded HLA binding prediction tool. The T-cell epitope and HLA ligand sequences are hyperlinked to T cell epitope or HLA ligand record tables. Most of the epitope and HLA-ligand records were enriched with additional annotation, e.g. ‘defined in healthy donors’, ‘associated with virus clearance’, ‘defined in cervical intraepithelial neoplasia (CIN) or cervical cancer patients’ and ‘defined by mass spectrometry from cervical cancer biopsy specimens’. Most of these peptides were derived from the two oncogenic proteins E6 and E7 of HPV16.

**Figure 4.**
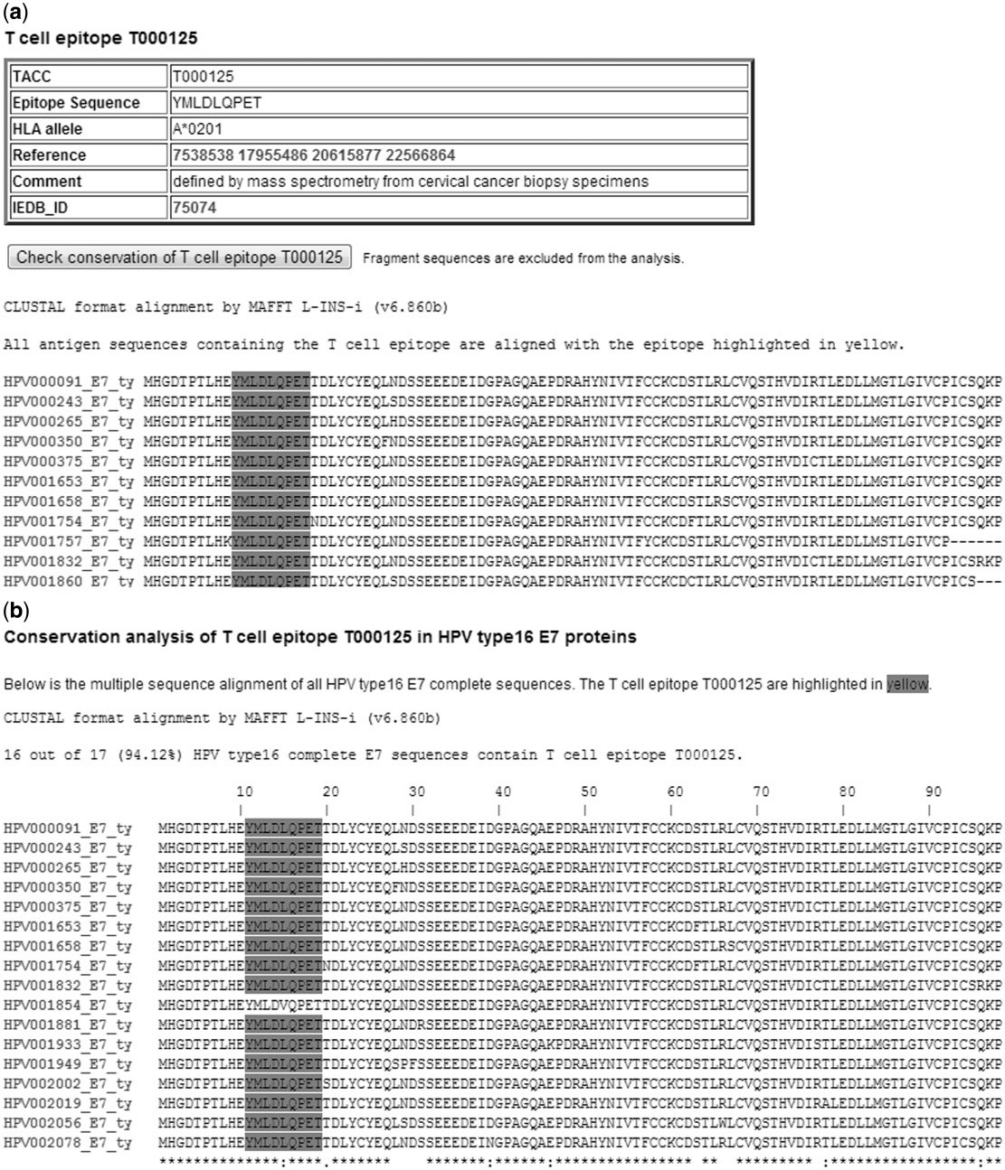
(**A**) A screenshot of a T cell epitope record table in the HPVdb. This table catalogs the relevant information of T cell epitope T000125, i.e. epitope sequence, restricted HLA allele, PubMed id(s) of the reference paper(s) and its characteristics (e.g. information on how the epitope was identified). A multiple sequence alignment of the protein sequences containing the epitope (highlighted) is displayed. (**B**) A screenshot of the conservation analysis result page obtained by clicking on ‘check conservation of T cell epitope T000125’ button.

Users can also search T cell epitopes and HLA ligands by keywords, such as epitope/ligand sequences or HLA allele names. Figure 4A is a screen shot of a T cell epitope record table—an A*0201 restricted T cell epitope in HPV type 16 E7 protein sequences. We can learn how conserved the epitope YMLDLQPET is by clicking on the button ‘check conservation of T cell epitope T000125’. As shown in Figure 4B, the epitope exists in all but one HPV type 16 E7 proteins (94.12% conserved).

**Figure 5.**
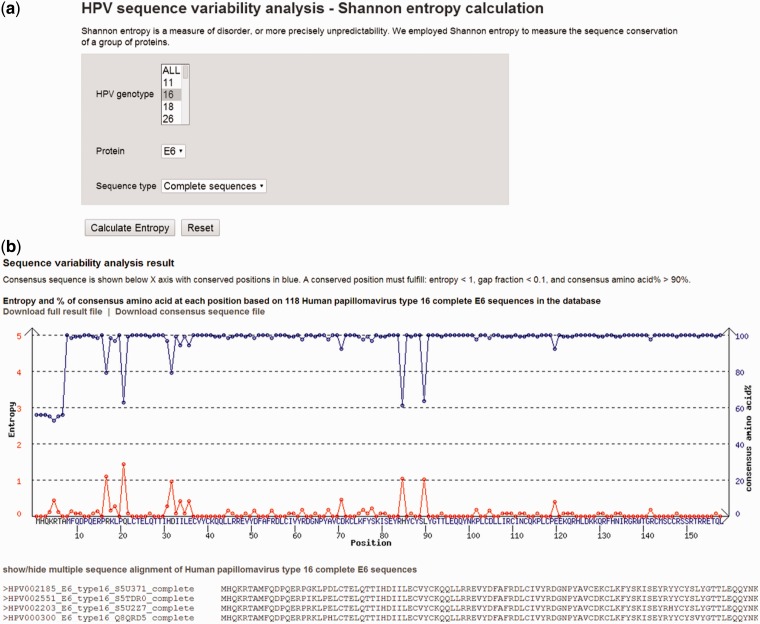
(**A**) A screenshot of sequence variability analysis tool page. (**B**) Plot of entropy (red curve) and percentage of sequences (blue curve) containing the consensus amino acid. The consensus sequence is shown below X-axis with conserved positions in blue. A conserved position is one with: entropy <1, gap fraction <0.1 and consensus amino acid >90%.

Figure 5A shows the sequence variability analysis tool page. The search parameters include virus genotype 16, protein E7 and complete sequence type. Figure 5B shows the sequence variability analysis result page. On top of the page, there is a plot of entropy (red curve) and percentage of sequences (blue curve) containing the consensus amino acid at position. The consensus sequence is shown below X-axis with conserved positions in blue. A conserved position is one with entropy <1, gap fraction <0.1 and consensus amino acid >90%. The detailed position-by-position amino acid variability information and the consensus sequence are available for download.

BLAST was integrated into the database to enable sequence similarity search. It can be used for protein and genotype identification. The MSA can be performed by selecting a protein name and virus genotype for sequence diversity analysis. The visualization tool provides three peptide display formats for mapping T cell epitopes or HLA ligands. In format 1, T cell epitope and HLA ligands are highlighted on the peptide sequences. In format 2, the peptides are highlighted in the MSA. In format 3, each peptide is shown in a separate line based on the restricting HLA alleles.

Table 2 summarizes the analysis tools integrated in the HPVdb and their uniform resource locators (URLs) to access them directly. More information of the HPVdb and instructions for using the database are available at http://cvc.dfci.harvard.edu/hpv/HTML/help.php.

**Table 2.** The analysis tools integrated in HPVdb and their URLs

Tool	URL	References
BLAST	http://blast.ncbi.nlm.nih.gov/Blast.cgi	(18)
MAFFT MSA	http://www.ebi.ac.uk/Tools/msa/mafft/	(19)
NetMHCpan	http://www.cbs.dtu.dk/services/NetMHCpan/	(20)
NetMHCIIpan	http://www.cbs.dtu.dk/services/NetMHCIIpan/	(21)
Search tool for HPV antigens	http://cvc.dfci.harvard.edu/hpv/HTML/search.php	
Search tool for T cell epitope/HLA ligand	http://cvc.dfci.harvard.edu/hpv/HTML/searchT.php	
Blast HPVdb	http://cvc.dfci.harvard.edu/hpv/HTML/blast.php	
MSA of HPV sequences	http://cvc.dfci.harvard.edu/hpv/HTML/alignment.php	
Sequence variability analysis tool	http://cvc.dfci.harvard.edu/hpv/HTML/varability.php	(14)
T cell epitope/HLA ligand visualization tool	http://cvc.dfci.harvard.edu/hpv/HTML/viewEpitope.php	
Classification of the viruses based on cancer risk	http://cvc.dfci.harvard.edu/hpv/HTML/classification.php	
HLA binding prediction tool	Embedded in each antigen entry table	
T cell epitope/HLA ligand conservation analysis tool	Embedded in each experimentally validated T cell epitope/HLA entry table; also embedded in each HLA binding prediction result page.	

## A Case Study—The Identification of a Conserved HPV16 E7 T Cell Epitope

Because HPV proteins E6 and E7 are functionally required for cancer initiation and persistence, they offer exceptional targets for immune-based therapies. We have identified, by mass spectrometry, an E7-derived cytotoxic T cell epitope (E7_11–19_) that was presented by cervical cancer cell lines as well as seven of nine HPV16-positive primary tumor cervical cancer biopsy samples (25–27). Our analyses have shown that the number of distinct cytotoxic T lymphocyte epitope targets on a tumor is small, requiring precise focusing of vaccine formulation. In the study, we identified the E7_11__–__19_, but not the related E7_11__–__20_ peptide, on all of the established HPV16 tumor cell lines (25). The latter peptide, which has received considerable attention in the literature as a possible tumor antigen, was incorrectly selected for vaccine formulation and was clinically ineffective (28). The two patients that did not display E7_11–19_ peptide also had a loss of the human cellular thioreductase GILT protein. Thioreductases are proteins that unfold cysteinylated proteins such as HPV E7, which was associated with altered HPV peptidome display in HPV16-driven cervical cancers (26).

The HPVdb played an important role to facilitate and speed up the study. First, *in silico* predictions of A*0201 binding peptides (both 9- and 10-mers) were performed (prediction result is not shown). Based on the prediction results, we synthesized 21 peptides. A*0201 binding assay identified 10 of them as binding peptides. Interferon γ (IFN γ) ELISpot assay was used to test immune recognition of the 10 A*0201-binding peptides in peripheral blood mononuclear cells isolated from six A*0201-positive healthy donors. There are only two HPV peptides, E7_11–19_ and E6_29–38_, eliciting spot-forming unit numbers 4- to 5-fold over background in one donor. MS^3^ Poisson detection mass spectrometry identified that the peptide E7_11–19_ (refer to Figure 4A for the epitope’s information) is physically displayed on HPV16-transformed, A*0201-positive cells. Given the expression of E7_11–19_ on HPV-16 transformed or transfected cell lines, we would like to know whether known strains of HPV16 conserve this epitope. We performed the epitope conservation analysis using the analysis tool embedded in each HPVdb T cell epitope entry page as shown in Figure 4A. As shown in the analysis result page in Figure 4B, the epitope is conserved in 16 of 17 (94.12% conserved) HPV16 E7 complete sequences. A single substitution mutation L15V in HPV001854 (UniProt ID: C0KXQ5) resulted in the immune escape. Among the 35 HPV16 cervical cancer or cervicitis patients we analyzed, only a single patient sample contained the HPV001854 sequence variant. Conserved HPV T cell epitopes displayed by HPV transformed tumors such as E7_11__–__19_ may be the basis of a therapeutic T cell-based cancer vaccine.

## DISCUSSION

HPV infection is prevalent among sexually active population. However, >95% of infections are temporary and are promptly cleared by the immune system (29, 30). The regression of tumor lesions and persistent high-risk infections depend on strong localized antigen-specific T cell responses (31). HPV-associated cancers express viral oncoproteins, and they represent ideal ‘non-self’ cancer antigens for the development of a therapeutic cancer vaccine (11). One of the research interests of our group at the Dana-Farber Cancer Institute is the discovery of T cell-based HPV vaccine targets. We have identified, by mass spectrometry, an E7-derived cytotoxic T cell epitope (E7_11–19_) that was presented by cervical cancer cell lines as well as seven of nine HPV16-positive primary tumor cervical cancer biopsy samples. With the data analysis and knowledge discovery capacity provided by the HPVdb, this epitope was found to be highly conserved among HPV16 strains. The information and knowledge derived through the computational analysis enabled by the HPVdb directly added to the potential clinical value of this epitope as a vaccine candidate. It highlights the importance of organized epitope information across various HPV types and strains for researchers in the field.

Several data sources provide information on HPV genomic and protein sequences. The HPVdb (http://ncv.unl.edu/Angelettilab/HPV/Database.html) was constructed a decade ago and has not been actively maintained (32). The Papillomavirus Episteme (PaVE) hosts 241 annotated papillomavirus genomes, 2245 genes and regions, 2004 protein sequences and 47 protein structures (http://pave.niaid.nih.gov/) (33). The PaVE provides basic bioinformatics tools to facilitate keyword and BLAST search, MSA and protein structure viewing. An external L1-specific typing tool developed by Piet Maes *et al.* (34) are integrated to the database to predict whether a new isolate is different from other named viruses and meets the criteria for a new type. The Human Papillomavirus Proteome Database hosts genomic and proteomic information on 150 HPV strains and their 1036 protein sequences and 743 predicted structures (35). The HPVdb was developed with a different purpose in mind, to facilitate data mining for HPV vaccinology and immunology. The HPVdb tightly integrates curated data and information on both antigen sequences and immunological epitopes with tailored analysis tools to aid rational vaccine design by discovery of vaccine targets. It is a unique data mining system for knowledge discovery in HPV with applications in T cell immunology and vaccinology.

To support T cell epitope discovery and make use of existing information and knowledge, we developed KB-builder, a framework that streamlines the development and deployment of web-accessible immunological knowledge systems. The KB-builder framework aims to speed up the immunological research and vaccine design by providing specialist knowledge bases that host cleaned, well-annotated and structured data suitable for the discovery of new knowledge. We built several prototypes focusing mainly on viral and tumor antigens using this framework for immunological knowledge discovery. Knowledge bases generated using KB-builder enable data mining using defined workflows. User-friendly analysis tools can be used individually or as part of workflows. The main purpose of vaccine knowledge bases is to help identify key experiments and reduce the overall number of required experiments for vaccine research including the discovery and design.

Systematic discovery of HPV vaccine targets relies heavily on the availability of accurate, up-to-date and well-organized antigen data. HPV antigen data are available through publications, technical reports and databases. These data vary in granularity and quality and are in various formats. The extraction of knowledge from the data scattered around using data mining techniques remains a challenging task. HPVdb is a specialized bioinformatics database that tightly integrates the content (data) and analysis tools to enable the automation of complex queries and data mining. The HPVdb enables speed up of rational vaccine design by providing accurate and well-annotated data coupled with tailored computational analysis tools.
